# Association Between Blood Groups and COVID-19 CT Severity: A Retrospective Analysis From a Tertiary Care Center

**DOI:** 10.7759/cureus.46506

**Published:** 2023-10-04

**Authors:** Sri Vengadesh Gopal, Vivek Sanker, Saravanan Pandian, Thiruvalluvan Vignesh, Krishna Vardhan M S, Arun Tipandjan, Sharini Cadiravane

**Affiliations:** 1 Surgery, Indira Gandhi Medical College and Research Institute, Puducherry, IND; 2 General Surgery, Noorul Islam Institute of Medical Science (NIMS) and Research Foundation, Trivandrum, IND; 3 General Surgery, Indira Gandhi Medical College and Research Institute, Puducherry, IND; 4 General Medicine, Indira Gandhi Medical College and Research Institute, Puducherry, IND; 5 Radiology, Indira Gandhi Medical College and Research Institute, Puducherry, IND

**Keywords:** ct severity, pandemic, sars-cov-2, blood group, covid-19

## Abstract

Introduction: The COVID-19 infection can have varied severity; presenting symptoms include fever, coughing, headaches, sore throats, exhaustion, muscle aches, loss of taste or smell, rhinorrhea, stomach pain, diarrhea, and vomiting. In various parts of the world, including India, researchers have looked into the relationship between blood type and the severity of SARS-CoV-2 infection. The aim of the study is to investigate the relationship between COVID-19 infection severity and blood group.

Methodology: A total of 1,222 COVID-19 patients with real-time reverse transcription-polymerase chain reaction (RT-PCR) confirmation of being COVID-positive were included in the study. Mortality rates, demographic information, comorbid illnesses, epidemiological information, laboratory test results, and comorbid disorders were all retrieved. Each participant's RH type and Groups A, B, O, and AB were determined. IBM SPSS software version 26 (IBM Corp., Armonk, NY) was used for the statistical analysis. For a normal distribution, quantitative variables were shown as mean standard deviation (SD), and for a non-normal distribution, median (interquartile range (IQR)). Frequency and percentages were used to present qualitative characteristics.

Results: Out of the 1,222 patients included in the study, 369 were normal, 406 were mild, 317 were moderate, and 130 were severe based on COVID-19 CT severity scoring. Among the blood groups, O positive (+) was the most common with 503 (41.2%) study participants, and AB negative (-) was the least common with seven (0.6%) participants.

Discussion: In our study, comparing various blood groups, blood group O individuals have the highest risk of developing severe COVID-19 illness, and blood group AB individuals have a reduced risk. In terms of Rh status, patients who are Rh-positive are at increased risk of developing severe COVID-19 infection when compared with Rh- individuals. In the Indian population, blood group O is the commonest, and blood group AB is the least prevalent. Most of the individuals were Rh+, and the rest were Rh-. This is attributed to the increased infection rate in individuals with O+ blood type seen in our study when compared with other studies.

Conclusion: The findings indicated that individuals with blood groups A, B, and AB may be at a higher risk of severe COVID-19 infection, while blood group O might provide some protective effect. These results align with previous studies worldwide, suggesting that blood groups could influence the susceptibility to and severity of viral infections. The study emphasizes the need for further research with larger sample sizes and diverse populations to validate these findings and explore the underlying mechanisms.

## Introduction

Coronavirus disease (COVID-19) is a respiratory disease that has spread widely throughout the world and become a global pandemic. We are still understanding the viral behavior completely. The symptoms and their severity vary from person to person. People infected with the SARS-CoV-2 infection present with mild, moderate, and severe symptoms ranging from fever, cough, headache, sore throat, fatigue, muscle ache, loss of smell or taste, rhinorrhea, abdominal pain, diarrhea, and vomiting. A severe COVID-19 infection presents with acute respiratory distress, septic shock, and death. Risk factors for increased morbidity and mortality include old age, male gender, comorbid illnesses like diabetes and hypertension, and an impaired immune system. The role of the blood group in predicting the severity of many bacterial and viral infections, including Norwalk virus, hepatitis B virus, *Helicobacter pylori*, *Plasmodium falciparum*, and SARS CoV-1, has been widely noted in the literature [1,2]. This is due to the differences in the carbohydrate antigen present on the surface of the red blood cells, which alter the host's vulnerability to microorganisms and the immune system.

The association between blood group and severity of SARS-CoV-2 infection has been studied in some parts of the world and also in India [3,4]. Some studies have found that the severity of COVID disease is associated with blood groups A, B, and AB, and blood group O has some protective effects [5,6]. The protective role in blood type O individuals is due to the presence of anti-A antibodies, which inhibit the interaction of SARS-CoV protein with angiotensin-converting enzyme 2 (ACE-2)-expressing cells [7]. The purpose of the study is to find out the association between blood group and COVID-19 infection severity among individuals admitted to a COVID-19-designated tertiary care hospital in South India.

## Materials and methods

This is a retrospective investigation conducted at Indira Gandhi Medical College and Research Institute, Puducherry, a COVID-19-designated tertiary care hospital in South India, after approval from the ethical review board of our institute (ID: No. 331/IEC-32/IGMCRI/PP-10-2021). A total of 1,222 COVID-19 patients were included in this study from January 2021 to June 2021, and the demographic data, comorbid conditions, epidemiological data, laboratory tests, and mortality rates were extracted via the medical records department (MRD) of our institute. Every patient was confirmed positive for SARS-CoV-2 via real-time reverse transcription-polymerase chain reaction (RT-PCR).

The severity of the COVID-19 infection was classified as mild, moderate, and severe according to the Centers for Disease Control (CDC) guidelines. Individuals who had various signs and symptoms of COVID-19 (e.g., fever, cough, sore throat, headache, malaise, nausea, vomiting, loss of taste and smell, etc.) but did not have shortness of breath, dyspnea, or abnormal chest imaging were mild, and those showing evidence of a lower respiratory illness during clinical assessment or imaging and who had an oxygen saturation (SpO2) ≥ 94% were labeled as having moderate disease. Patients with SpO2 < 94% on room air, a respiratory rate of > 30 breaths/min, or > 50% lung infiltrates, and those requiring mechanical ventilation were labeled as severe. Patients admitted for observation for less than 24 hours, whose data were not available, who were not traceable, and in whom CT chest was not done, were excluded.

According to Pan et al.'s semi-quantitative CT severity scoring method, the following values were assigned to each of the five lung lobes, taking into consideration the extent of anatomical involvement [8, 9]: Each of the three right lung lobes and the two left lung lobes were assessed individually, and the percentages of lobe involvement were determined based on visual examination. The scoring system included score 1 (5% of the area involved), score 2 (5%-25% of the area involved), score 3 (25%-50% of the area involved), score 4 (50%-75% of the area involved), and score 5 (> 75% of the area involved) to categorize the visual severity of the chest CT. This resulted in a total score of 25.

By considering the percentage of each lobe's area affected in the CT scan, a severity score ranging from 0 (no involvement) to 25 (maximum involvement, when all five lobes have over 75% involvement) was calculated. The total CT score was obtained by summing the individual lobar scores. To grade the CT severity, the following criteria were used: mild (grade 1) (< 8), moderate (grade 2) (8-15), and severe (grade 3) (> 15).

In this study, we examined the ABO blood groups and Rh types of all participants. We conducted a statistical analysis using IBM SPSS software version 26 (IBM Corp., Armonk, NY). For quantitative variables, we expressed the data as mean ± standard deviation (SD) for normally distributed data and as median (interquartile range (IQR)) for non-normally distributed data. Qualitative variables were presented as frequencies and percentages.

To compare the four groups, we employed the Student's t-test for quantitative variables and the Chi-square test for qualitative variables. We also calculated the odds ratio (OR) and 95% confidence interval (CI) to assess the severity of COVID-19 disease, mortality, and hospital stay. Additionally, we depicted the correlation between the blood group and laboratory parameters through histograms distributed among the four groups.

Furthermore, we performed logistic regression to identify predictors of severe COVID-19 disease specifically related to ABO blood groups. We considered a p-value of less than 0.05 as statistically significant.

## Results

CT severity

Of the 1,222 patients included in the study, 369 (30.2%) had a normal score, 406 (33.2%) scored mild, 317 (24.9%) had a moderate score, and 130 (10.6%) patients scored severe based on COVID-19 CT severity scoring. Also, (Table 1).

**Table 1:** Frequency distribution of patients with COVID-19 CT severity scoring and their respective blood groups

Blood Group	CT Severity Scoring								Total (n=1222)
	Normal (n=369, 30.1%)		Mild (n=410, 33.5%)		Moderate (n=314, 25.7%)		Severe (n=130, 10.6%)		
	N	%	N	%	N	%	N	%	
A-	4	1.1	10	2.4	4	1.3	2	1.5	20
A+	32	8.7	86	21.0	67	21.3	23	17.7	207
B-	6	1.6	3	0.7	7	2.2	2	1.5	18
B+	114	30.9	123	30.0	83	26.4	35	26.9	355
O-	11	3.0	8	2.0	12	3.8	3	2.3	34
O+	170	46.1	150	36.6	124	39.5	59	45.4	503
AB-	1	0.3	3	0.7	3	1.0	0	0.0	7
AB+	31	8.4	27	6.6	14	4.5	6	4.6	78

Blood group

The most common blood group was O positive (+) with 503 (41.2%) participants, and AB negative (-) was the least common with seven (0.6%) participants. The frequency distribution of the other blood groups was as follows: A-: 20 (1.6%), A+: 207 (16.9%), B-: 18 (1.5%), B+: 355 (29.1%), O-: 34 (2.8%), and AB+: 78 (6.4%) (Table 1).

Blood group and COVID-19 CT severity

The frequency distribution of patients with various COVID-19 CT severity and their respective blood groups is shown in Table 1.

Chest CT severity and COVID-19 severity

Table 2 shows that there was a significant difference in the mean severity score among the various blood groups, with F = 2.580 and P = 0.012.

**Table 2:** The association between COVID-19 severity and CT chest severity

	Sum of Squares	Df	Mean Square	F	Sig.
Between Groups	18.389	7	2.627	2.580	.012
Within Groups	1235.917	1214	1.018		

The frequency distribution of patients with various COVID-19 CT severity and their respective severity categories is shown in Table 3.

**Table 3:** Frequency distribution of patients with various COVID-19 CT severities and their respective severity categories

	N	Mean	Std. Deviation	Std. Error	95% Confidence Interval for Mean	
					Lower	Upper
Mild	406	4.517	1.8213	.0904	4.340	4.695
Moderate	317	4.580	1.7889	.1005	4.383	4.778
Normal	369	5.073	1.5991	.0832	4.909	5.237
Severe	130	4.700	1.7238	.1512	4.401	4.999
Total	1222	4.721	1.7520	.0501	4.623	4.819

Table 4 shows that there was a significant difference in mean severity score among the various severity categories, with F = 7.608 and P = 0.0001.

**Table 4:** The association between blood groups and COVID-19 severity

	Sum of Squares	Df	Mean Square	F	Sig.
Between Groups	68.941	3	22.980	7.608	.000
Within Groups	3678.902	1218	3.020		
Total	3747.844	1221			

Table 5 shows that there was a significant correlation between the CT severity and various blood groups, with a Pearson's correlation coefficient of 0.096 and a p-value of 0.001.

**Table 5:** Correlation between the CT severity and the blood group categories **Correlation is significant at the 0.01 level (two-tailed).

Correlations			
		CT severity	Blood Group
CT severity	Pearson's Correlation	1	.096**
	Sig. (Two-tailed)		.001
	N	1222	1222
Blood Group	Pearson's Correlation	.096**	1
	Sig. (Two-tailed)	.001	
	N	1222	1222

## Discussion

Many studies have individually investigated the association of blood groups with infection and the severity of infection based on CT [10]. We investigated whether there is an association between the blood group and the severity of the COVID-19 infection based on CT. In our study, we found that there is a significant association between blood groups and COVID-19 CT severity. The characteristic features of COVID-19 in chest CT are ground glass opacity, consolidation, crazy paving pattern, pulmonary vascular enlargement, and linear opacification [11]. Based on CT findings, we categorized the patients into mild, moderate, and severe [12].

Among 1,222 patients with RT-PCR-proven COVID-19 infection, 10% were A-, 11.1% were A+, 11.1% were B+, 9.8% were B+, 8.8% were O+, and 11.7% were O-. Among the study participants, 0% of AB- and 7.6% of AB+ patients were found to have severe cases. In our study, compared with various blood groups, blood group O individuals have the highest risk of developing severe COVID-19 illness, and blood group AB individuals have a reduced risk. In terms of Rh status, patients who are Rh+ are at increased risk of developing severe COVID-19 infection when compared with Rh- individuals. The findings are not congruent with the study conducted by Zhao et al., where they showed that blood group O is associated with a lower risk of infection and death, and an increased infection rate and death are seen in blood group A [13]. The increased susceptibility of hosts to infection in blood type A individuals is also reported in other studies [3,14]. The protective role seen in Rh- individuals is also demonstrated in other studies [15,16]. Another study also demonstrated the protective role of the Rh- factor; in addition, they reported that blood group O individuals have lower odds of testing positive, and AB+ individuals have higher odds of testing positive [5].

It is also shown that blood type B is associated with an increased infection rate in the Asian population [16]. A study by Hoiland et al. reported that AB+ individuals have the highest risk of mechanical ventilation due to increased hepatic and renal biomarkers when compared with blood group O and B individuals, which is divergent from the findings of our study [17]. A similar study was done among the Indian population, and the severity was assessed based on COVID-19 infection mortality, revealing the protective role of blood type O individuals against COVID-related deaths [4]. This protective role in blood type O individuals was explained in other studies [18]. The blood type O and Rh- individuals have a lower risk of infection, but the O- blood type has no protective effect against COVID-19 illness and mortality [19].

In the Indian population, blood group O is the commonest, and blood group AB is the least prevalent. Most of the individuals are Rh+, with the rest being Rh- [20]. Sociodemographic details and comorbidities may influence the susceptibility of the host to infection. This is attributed to the increased infection rate in individuals with O+ blood type as seen in our study when compared with other studies.

Previous studies have described the glycan structures at different N-glycosylation sites of the SARS-CoV S protein. Recently, the N-glycans of the recombinant SARS-CoV-2 S protein were characterized, although ABH antigen structures were not included, possibly due to the cell line used for protein production. Interestingly, the receptor-binding domains of SARS-CoV-2 and SARS-CoV S proteins share structural similarities, and the glycosylation of S trimers covers the receptor-binding domains. This raises the possibility that human anti-A antibodies could specifically bind to the SARS-CoV-2 S protein, potentially blocking its interaction with the angiotensin-converting enzyme 2 receptor (ACE2R) and preventing entry into lung epithelial cells. In support of this hypothesis, both monoclonal and naturally occurring anti-A antibodies have been shown to inhibit the interaction between the SARS-CoV S protein and ACE2R when the A antigen is associated with the spike (S) protein. Although not definitive, current evidence supports this mechanism for SARS-CoV-2 as well. Furthermore, there is emerging evidence suggesting that the receptor-binding domain of SARS-CoV-2 shares sequence similarity with a lectin family known to bind blood group antigens, which could explain the association between blood group A and SARS-CoV-2 [7,21-27].

**Figure 1:**
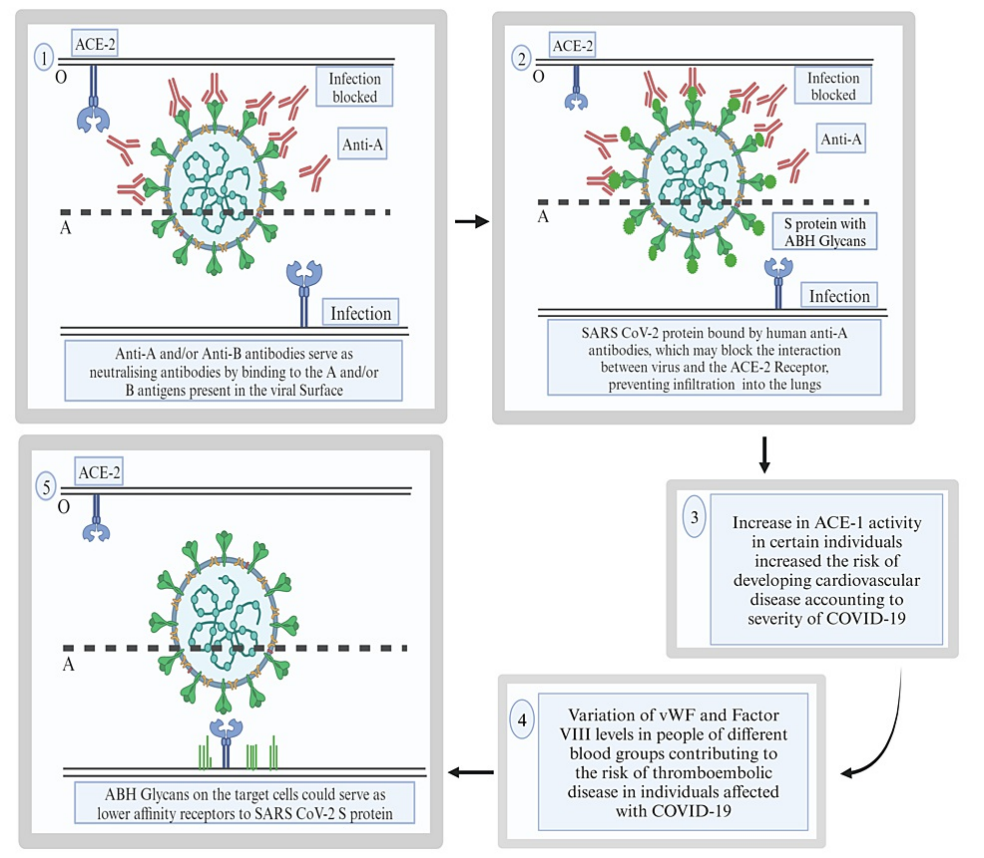
Proposed mechanisms and theoretical pathways suggest the interaction of the blood group and SARS-CoV-2, accounting for the severity of the disease. The image has been created by the authors. ACE-2: angiotensin-converting enzyme 2; S protein: spike protein; ACE-1: angiotensin-converting enzyme 1; vWF: Von Willebrand factor

Another potential explanation for the association between group A and severe COVID-19 is an increase in angiotensin-converting enzyme 1 (ACE-1) activity, which may predispose individuals to cardiovascular complications. Additionally, higher levels of Von Willebrand factor (vWF) and factor VIII in group A individuals, along with the acute phase reaction induced by infection, could contribute to severe outcomes (Figure 1).

However, it should be noted that the variability in anti-A and anti-B antibody titers among individuals, as well as the significantly higher binding affinity of SARS-CoV-2 S protein for ACE2R compared to SARS-CoV, may complicate the interpretation of large population studies and the potential neutralizing effect of these antibodies [28-30].

Limitations 

The available research on the association of blood groups with the severity of infection in COVID-19 patients and its implications for our local population is currently limited. Conducting a randomized study with appropriate control groups to compare the outcomes and effects of blood groups in COVID-19 patients would provide valuable insights. However, due to the lack of extensive research in our specific population, it is challenging to draw definitive conclusions or make direct comparisons with control groups. Further studies specifically focused on our population would be valuable in gaining a better understanding of this phenomenon and optimizing patient management and outcomes.

## Conclusions

This retrospective study conducted at a COVID-designated tertiary care hospital in South India examined the relationship between blood group and the severity of COVID-19 infection. The study included 1,222 COVID-19 patients, and various factors such as demographic data, comorbid conditions, laboratory tests, and mortality rates were analyzed. The findings indicated that individuals with blood groups A, B, and AB may be at a higher risk of severe COVID-19 infection, while blood group O might provide some protective effect. These results align with previous studies worldwide, suggesting that blood groups could influence the susceptibility to and severity of viral infections. The study emphasizes the need for further research with larger sample sizes and diverse populations to validate these findings and explore the underlying mechanisms. Understanding the role of blood groups in COVID-19 can assist in risk stratification, public health interventions, and targeted therapeutic approaches for improved disease outcomes.
